# Synchronous deficits in cumulative muscle protein synthesis and ribosomal biogenesis underlie age‐related anabolic resistance to exercise in humans

**DOI:** 10.1113/JP272857

**Published:** 2016-11-07

**Authors:** Matthew S. Brook, Daniel J. Wilkinson, William K. Mitchell, Jonathan N. Lund, Bethan E. Phillips, Nathaniel J. Szewczyk, Paul L. Greenhaff, Kenneth Smith, Philip J. Atherton

**Affiliations:** ^1^ MRC‐ARUK Centre of Excellence for Musculoskeletal Ageing ResearchClinical, Metabolic and Molecular PhysiologyUniversity of NottinghamDerbyUK; ^2^ Departments of SurgeryRoyal Derby HospitalDerbyUK

**Keywords:** ageing, exercise, hypertrophy, muscle, protein synthesis, ribosomal biogenesis, signalling, stable isotope

## Abstract

**Key points:**

- Resistance exercise training (RET) is one of the most effective strategies for preventing declines in skeletal muscle mass and strength with age.
- Hypertrophic responses to RET with age are diminished compared to younger individuals.
- In response to 6 weeks RET, we found blunted hypertrophic responses with age are underpinned by chronic deficits in long‐term muscle protein synthesis.
- We show this is likely to be the result of multifactorial deficits in anabolic hormones and blunted translational efficiency and capacity.
- These results provide great insight into age‐related exercise adaptations and provide a platform on which to devise appropriate nutritional and exercise interventions on a longer term basis.

**Abstract:**

Ageing is associated with impaired hypertrophic responses to resistance exercise training (RET). Here we investigated the aetiology of ‘anabolic resistance’ in older humans. Twenty healthy male individuals, 10 younger (Y; 23 ± 1 years) and 10 older (O; 69 ± 3 years), performed 6 weeks unilateral RET (6 × 8 repetitions, 75% of one repetition maximum (1‐RM), 3 times per week). After baseline bilateral vastus lateralis (VL) muscle biopsies, subjects consumed 150 ml D_2_O (70 atom%; thereafter 50 ml week^−1^), further bilateral VL muscle biopsies were taken at 3 and 6 weeks to quantify muscle protein synthesis (MPS) via gas chromatography–pyrolysis–isotope ratio mass spectrometry. After RET, 1‐RM increased in Y (+35 ± 4%) and O (+25 ± 3%; *P* < 0.01), while MVC increased in Y (+21 ± 5%; *P* < 0.01) but not O (+6 ± 3%; not significant (NS)). In comparison to Y, O displayed blunted RET‐induced increases in muscle thickness (at 3 and 6 weeks, respectively, Y: +8 ± 1% and +11 ± 2%, *P* < 0.01; O: +2.6 ± 1% and +3.5 ± 2%, NS). While ‘basal’ longer term MPS was identical between Y and O (∼1.35 ± 0.1% day^−1^), MPS increased in response to RET only in Y (3 weeks, Y: 1.61 ± 0.1% day^−1^; O: 1.49 ± 0.1% day^−1^). Consistent with this, O exhibited inferior ribosomal biogenesis (RNA:DNA ratio and c‐MYC induction: Y: +4 ± 2 fold change; O: +1.9 ± 1 fold change), translational efficiency (S6K1 phosphorylation, Y: +10 ± 4 fold change; O: +4 ± 2 fold change) and anabolic hormone milieu (testosterone, Y: 367 ± 19; O: 274 ± 19 ng dl^−1^ (all *P* < 0.05). Anabolic resistance is thus multifactorial.

## Key points

- Resistance exercise training (RET) is one of the most effective strategies for preventing declines in skeletal muscle mass and strength with age.
- Hypertrophic responses to RET with age are diminished compared to younger individuals.
- In response to 6 weeks RET, we found blunted hypertrophic responses with age are underpinned by chronic deficits in long‐term muscle protein synthesis.
- We show this is likely to be the result of multifactorial deficits in anabolic hormones and blunted translational efficiency and capacity.
- These results provide great insight into age‐related exercise adaptations and provide a platform on which to devise appropriate nutritional and exercise interventions on a longer term basis.

**Abbreviations**
1‐RM: one repetition maximum
ABR: absolute breakdown rate
Akt: protein kinase B
APE: atom percent excess
ASP: alkali soluble protein
ASR: absolute synthetic rate
BMI: body mass index
D_2_O: deuterium oxide
DXA: dual energy X‐ray absorptiometry
4EBP: eukaryotic translation initiation factor 4E‐binding protein 1
eEF2: eukaryotic elongation factor 2
ERK: extracellular signal‐regulated kinase
FBR: fractional breakdown rate
FFM: fat‐free mass
FC: fold change
FGR: fractional growth rate
FSR: fractional synthetic rate
GnRH: gonadotropin‐releasing hormone
IGF‐1: insulin‐like growth factor 1
*L*_f_: fibre length
MAFbx: muscle atrophy F‐box
MET: metabolic equivalent
MPB: muscle protein breakdown
MPS: muscle protein synthesis
MT: muscle thickness
MuRF1: muscle RING finger 1
MVC: maximum voluntary contraction
mTORC1: mechanistic target of rapamycin complex 1
PCA: perchloric acid
P70S6K1: ribosomal protein S6 kinase 1
RB: retinoblastoma
rDNA: ribosomal DNA
RE: resistance exercise
RET: resistance exercise training
rps6: ribosomal protein S6
SMI: skeletal muscle index
T: trained
TIF1a: transcription initiation factor 1
TFFM: thigh fat‐free mass
θ: pennation angle
UBF: upstream binding factor 1
UT: untrained
VL: vastus lateralis

## Introduction

Advancing age is associated with incipient declines in skeletal muscle mass, strength and quality, processes termed sarcopenia and dynapenia (Mitchell *et al*. 2012). Since skeletal muscles are central for locomotion and the performance of everyday habitual activities, declines in muscle mass and function lead to impaired quality of life and independence. Furthermore, as skeletal muscle plays a substantial role in whole body metabolic health, muscle loss results in decreased metabolic rate (Tzankoff & Norris, 1977) and increased risk of morbidity (Luukinen *et al*. 1997) and mortality (Laukkanen *et al*. 1995). With increases in life expectancy showing no signs of slowing (Christensen *et al*. 2009) and those > 60 years predicted to represent 25% of the population by 2050, increased strains on the healthcare system are expected to rise (Janssen *et al*. 2004).

Muscle mass is controlled by the diurnal balance between muscle protein synthesis (MPS) and muscle protein breakdown (MPB) (Atherton & Smith, 2012). Therefore, declines in mass occur when MPS is chronically lower than MPB. On a diurnal basis, the most significant regulators of MPS and MPB are food intake (in particular protein; Atherton *et al*. 2010; Bukhari *et al*. 2015; Wilkinson *et al*. 2015) and physical activity (Kumar *et al*. 2009 *a*, 2012; Breen *et al*. 2013; Wilkinson *et al*. 2014). Both feeding and exercise transiently stimulate MPS, and when combined result in sustained positive net balance and eventually protein accretion, i.e. following a resistance exercise training (RET) programme (Phillips *et al*. 1997; Moore *et al*. 2009, 2015; Fry *et al*. 2011; Bukhari *et al*. 2015). Whilst basal rates of MPS and MPB are not detectably lower in old age (Volpi *et al*. 2001; Kumar *et al*. 2009 *b*), older individuals do exhibit ‘anabolic resistance’, manifesting as a reduced anabolic response to these key growth signals regulating homeostasis (Cuthbertson *et al*. 2005; Kumar *et al*. 2009 *b*; Fry *et al*. 2011; Wall *et al*. 2015). Over time, inadequate replenishment of muscle protein lost during fasting or inactivity leads to a progressive loss of muscle with age.

RET arguably offers the most effective countermeasure and lowest risk profile strategy to mitigate age‐related declines in skeletal muscle mass and strength (Fiatarone *et al*. 1994). However in response to the same RET programme, older individuals generally show attenuated muscle hypertrophy (Welle *et al*. 1996; Kosek *et al*. 2006; Greig *et al*. 2011; Peterson *et al*. 2011; Mero *et al*. 2013), although equal adaptations have been reported (Häkkinen *et al*. 1998 *b*; Mayhew *et al*. 2009). The exact mechanisms leading to RET‐induced anabolic resistance are unclear. Ageing can be associated with neuromuscular deficits (e.g. loss of motor units; Campbell *et al*. 1973), sustained malnutrition (Morley, 2012), physical inactivity (Troiando *et al*. 2008), suboptimal anabolic hormonal profiles (Feldman *et al*. 2002) and various co‐morbidities and chronic diseases (Marengoni *et al*. 2011), all of which theoretically represent unfavourable attributes for hypertrophy. Additionally at the cellular level, muscle signalling responses linked with increased translational efficiency (e.g. mechanistic target of rapamycin complex 1 (mTORC1) signalling) and capacity (ribosomal biogenesis) have been shown to be blunted ‘acutely’ with ageing (Kumar *et al*. 2009 *a*; Fry *et al*. 2011; Stec *et al*. 2015). Nonetheless, the coordinate mechanisms remain elusive and are likely to be multifactorial in nature.

Current assumptions on the mechanisms of muscle hypertrophy in youth and ageing have been largely derived from ‘acute’ metabolic and molecular studies, i.e. those undertaken after a single bout of resistance exercise (RE)(Miller *et al*. 2005; Kumar *et al*. 2009 *b*; Fry *et al*. 2011; Bukhari *et al*. 2015). Nonetheless, due to the disparity in acquisition of these acute response, i.e. nutritional provision, length of measurement and exercise paradigms, the exact relationships between acute responses and blunted adaptations are unclear (Mitchell *et al*. 2014; Atherton *et al*. 2015). Similar issues are associated with RET studies where hypertrophic adaptations are assessed with a single beginning and end‐point (Häkkinen *et al*. 1998 *b*; Mayhew *et al*. 2009) in which physiological insight is great but metabolic and molecular insight is limited. To circumvent these limitations, we quantified MPS responses to 6 weeks RET in young and older individuals using novel heavy water, deuterium oxide (D_2_O) techniques (Robinson *et al*. 2011; Wilkinson *et al*. 2014); simultaneously we temporally assessed indices of cellular translational efficiency and capacity. We hypothesized that following 6 weeks of RET, skeletal muscle hypertrophy would be blunted with age, primarily due to long‐term deficits in cumulative MPS responses with the underlying mechanisms multifactorial.

## Methods

### Subject characteristics and ethics

Ten healthy younger (23 ± 1 years, BMI: 24 ± 1) (Brook *et al*. 2015) and older (69 ± 1 years, BMI 25.8 ± 1) men were recruited. Volunteers were screened by medical questionnaire, physical examination, and resting electrocardiogram, with exclusions for metabolic, respiratory and cardiovascular disorders or any other symptoms of ill health. Subjects had clinically normal blood chemistry, were normotensive (<140/90), and were not prescribed any medications: all subjects performed activities of daily living and recreation but did not undertake in any RET other than that described in the study and had not participated in any RET within the last 12 months. All subjects provided their written, informed consent to participate after all procedures and risks (in relation to muscle biopsies, blood sampling, etc.) were explained. This study was approved by The University of Nottingham Ethics Committee, with all studies conducted according to the declaration of Helsinki and preregistered (clinicaltrials.gov registration no. NCT02152839).

### Conduct of the study

**Figure 1:**
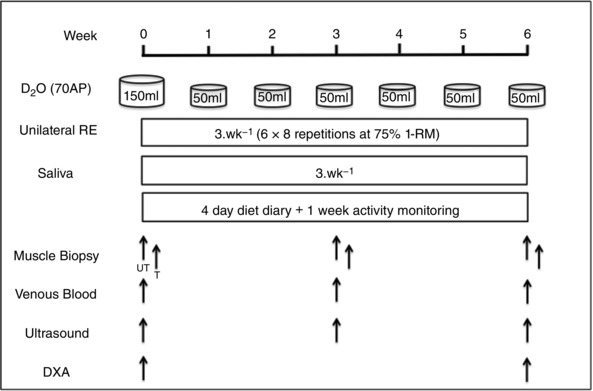
Schematic diagram of study protocol

This study involved a bilateral (i.e. two legged) protocol whereby one leg was used as an untrained (UT) internal time control with the other leg acting as the RET (trained; T) leg, with the dominant leg assigned as T. Following inclusion to the study, subjects were studied over a 6‐week period. On the first day of study, subjects arrived at the laboratory at 08.30 h following an overnight fast. Following assessment of vastus lateralis (VL) muscle architecture by ultrasound (Mylab 70; Esaote Biomedica, Italy) and body composition by dual energy X‐ray absorptiometry (DXA; Lunar Prodigy II, GE Medical Systems, Little Chalfont, UK), subjects completed the first session of RET consisting of unilateral knee‐extension exercise (i.e. 6 × 8 repetitions at 75% of one repetition maximum (1‐RM)). Bilateral biopsies were taken from the VL muscle 60–90 min (75 ± 2 min) after unilateral exercise under sterile conditions, using the conchotome biopsy technique (Dietrichson *et al*. 1987) with 1% lidocaine (B. Braun, Melsungen, Germany) as local anaesthetic. Muscle was rapidly dissected free of fat and connective tissue, washed in ice‐cold phosphate‐buffered saline (PBS), and then frozen in liquid N_2_ and stored at –80°C until further analysis. Immediately post‐RET, subjects provided a saliva sample (collected in sterile plastic tubes) and consumed a 150 ml bolus of D_2_O (70 atom%; Sigma‐Aldrich, Poole, UK), with the aim to label the body water pool to ∼0.2% atom percent excess (APE), which was maintained in a pseudo‐steady state with weekly top‐up boluses (50 ml week^−1^). In addition, venous blood samples were collected into lithium heparin coated tubes, immediately cold centrifuged at 1750 *g*, with plasma fractions aliquoted and frozen at –80°C until analysis. Thereafter, subjects returned to the lab 3 times per week to undertake supervised unilateral RET with 1‐RM assessments of the T leg every ∼10 days to ensure progressive intensity. Further bilateral muscle biopsies (∼90 min after RET in order to investigate the temporal nature of acute anabolic signalling responses to progressive RET), ultrasound measures of muscle architecture and venous blood samples were taken at 3 and 6 weeks and DXA performed at 6 weeks. For the temporal monitoring of body water enrichment, each participant provided a saliva sample on RET‐visits > 30 min after their last meal or drink, with extra samples taken ∼3 h after weekly 50 ml boluses to ensure body water enrichment was accurately represented. These were collected in sterile plastic tubes and immediately cold‐centrifuged at 16,000 *g* to remove any debris that might be present; they were then aliquoted into 2 ml glass vials and frozen at –20°C until analysis. To measure levels of physical activity, subjects wore combined heart rate and activity monitors (Actiheart, CamMtech Ltd, Cambridge, UK) for 1 week during the study. Actiheart data were checked for missing or extrapolated values; ≥ 80% of minute‐by‐minute physical activity data had to be available for a 24 h recording to be accepted, 85% of subjects had at least ≥ 4 days of data with the remaining having ≥ 1 day of data that were included in the analysis.  Dietary intake was monitored by completion of 4‐day diet diaries during the study and was analysed using Microdiet (Downlee Systems Ltd, Chapel‐en‐le‐Frith, UK). A detailed representative schematic diagram of the study protocol in its entirety is depicted in Fig. 1.

### Muscle functional tests

Basal measures of maximal voluntary contraction (MVC) and 1‐RM were ascertained in the week prior to the first study day. MVC was measured with isometric contractions conducted in a sitting position using an isokinetic dynamometer (Isocom; Isokinetic Technologies, Eurokinetics, UK) over a range of four knee joint angles (90–60 deg), with full extension corresponding to 0 deg. Subjects were seated in the dynamometer chair and secured into position using a chest strap. Contractions lasted 4 s, with a 30 s rest period between contractions and 90 s between knee joint angles. Unilateral leg extension 1‐RM was assessed on the dominant leg (Technogym, Gambettola, Italy). After the procedure was explained, subjects performed a light warm up to avoid injury and ensure familiarity whilst avoiding fatigue; 1‐RM was then achieved (20 on Borg's rating of perceived exertion scale) in as few repetitions as possible with a maximum of five repetitions. The first repetition was estimated at 50% 1‐RM and then increased until subjects could not complete controlled contraction throughout the range of motion, with 3 min in‐between attempts. Functional tests were repeated throughout (about every 10 days), with each set of tests performed on a non‐biopsy day to avoid influencing subsequent measures.

### Muscle architecture and DXA derived mass

Muscle architecture was measured as described (Franchi *et al*. 2014). Briefly, the architectural parameters muscle thickness (MT), fibre length (*L*_f_) and pennation angle (θ) were quantified, by the same ‘blinded’ investigator (M.B.), from ultrasound scans using ImageJ 1.42q (National Institutes of Health, Bethesda, MD, USA). The visible portion of the *L*_f_ was assessed directly using this software with MT measured as the distance between the superficial and deep aponeuroses. θ was measured as the intersection between fascicles and the deep tendon aponeurosis. DXA‐derived thigh fat‐free mass (TFFM) was determined from the lowest point of the ischium to the knee space.

### Body water and protein bound alanine muscle fraction enrichment

Body water and muscle protein enrichment were measured as previously described (Wilkinson *et al*. 2014). Briefly, pure fractions of body water were extracted by heating 100 μl of saliva in an inverted 2 ml autosampler vial for 4 h at 100°C. Vials were then placed upright on ice to condense extracted body water and transferred to a clean autosampler vial ready for injection. Body water (0.1 μl) was injected into a high‐temperature conversion elemental analyser (Thermo Finnigan, Thermo Scientific, Hemel Hempstead, UK) connected to an isotope ratio mass spectrometer (Delta V Advantage, Thermo Scientific). For isolation of myofibrillar protein, 30–50 mg of muscle was homogenized in ice‐cold homogenization buffer (Wilkinson *et al*. 2014), rotated for 10 min, and the supernatant was collected after centrifugation at 13,000 *g* for 5 min at 4°C. The myofibrillar pellet was solubilized in 0.3 m NaOH and separated from the insoluble collagen by centrifugation, and the myofibrillar protein was precipitated using 1 m perchloric acid (PCA). Sarcoplasmic proteins were precipitated from the sample homogenate with 1 m PCA and separated by centrifugation. Protein‐bound amino acids were released using acid hydrolysis by incubating at 110°C in 0.1 m HCl in Dowex H^+^ resin slurry overnight before being eluted from the resin with 2 m NH_4_OH and evaporated to dryness; amino acids were then derivatized as their *n*‐methoxycarbonyl methyl esters. Dried samples were suspended in 60 μl distilled water and 32 μl methanol, and following vortex, 10 μl of pyridine and 8 μl of methylchloroformate were added. Samples were vortexed for 30 s and left to react at room temperature for 5 min. The newly formed *n*‐methoxycarbonyl methyl esters of amino acids were then extracted into 100 μl of chloroform. A molecular sieve was added to each sample for ∼20 s before being transferred to a clean glass Gas Chromatography insert, removing any remaining water by size exclusion absorption. Incorporation of deuterium into protein bound alanine was determined by gas chromatography–pyrolysis–isotope ratio mass spectrometry (Delta V Advantage) alongside a standard curve of known l‐alanine‐2,3,3,3‐d4 enrichment to validate measurement accuracy of the instrument.

### Calculation of fractional synthetic rate

Myofibrillar MPS was calculated from incorporation of deuterium‐labelled alanine into protein, using the enrichment of body water (corrected for the mean number of deuterium moieties incorporated per alanine, 3.7, and the dilution from the total number of hydrogens in the derivative, i.e. 11) as the surrogate precursor labelling between subsequent biopsies. Fractional synthetic rate (FSR) is as follows:

FSR =− ln −1( APE  Ala )( APE P)t,

where APE_Ala_ is deuterium enrichment of protein‐bound alanine, APE_P_ is mean precursor enrichment over the time period, and *t* is the time between biopsies. Thigh absolute synthetic rate was calculated as:

ASR = FSR 100× TFFM ×( ASP )100,

where TFFM is thigh fat‐free mass derived by DXA and alkali soluble protein (ASP) is the average soluble protein over the 6 weeks. Absolute protein breakdown rate (ABR) was calculated as:

ABR = FBR 100× TFFM ×( ASP )100,

where fractional breakdown rate (FBR) is calculated as  FBR = FSR − FGR , with the fractional growth rate (FGR) assumed to be linear over 6 weeks. Using a linear growth rate takes into account the average adaptation in growth and turnover rates over the 6 weeks; however, this limits the ability to investigate temporal changes in absolute rates.

### Muscle protein, DNA and RNA concentrations (translational efficiency/capacity)

To determine muscle alkaline soluble protein, DNA and RNA concentrations (per mg dry weight), ∼15 mg muscle tissue was freeze dried. Dry tissue was homogenized in 0.2 m PCA. Following centrifugation at 11,680 *g* and washing with 0.2 m PCA, the resulting pellet was resuspended in 0.3 m NaOH and alkali soluble proteins were quantified by spectrophotometry (NanoDrop Lite, Thermo Scientific). Thereafter proteins were precipitated with 1 m PCA before centrifugation and removal of the supernatant for RNA quantification by UV spectrophotometry. The remaining pellet was resuspended in 2 m PCA and incubated at 70°C for 1 h before centrifugation and removal of the supernatant for quantification of DNA by spectrophotometry (Laurent *et al*. 1978; Smith *et al*. 2011).

### Immunoblotting for Akt–mTORC1 signalling components and ELISA

Immunoblotting was performed on VL muscle sarcoplasmic fractions with protein concentrations determined by spectrophotometry (NanoDrop Lite); samples were standardized to 1 mg ml^−1^ by dilution with 3× Laemmli loading buffer and heated at 95°C for 5 min. Precisely 15 μg of proteins were loaded onto Criterion XT Bis–Tris–12% SDS‐PAGE gels (Bio‐Rad) for electrophoresis at 200 V for 1 h. As previously described (Atherton *et al*. 2009; Crossland *et al*. 2013) samples were transferred to polyvinylidene difluoride membranes for 45 min at 100 V. Membranes were subsequently blocked in 2.5% low‐fat milk (diluted in Tris‐buffered saline and 0.1% Tween‐20 (TBS‐T)) for 1 h at ambient temperature and then incubated rocking overnight at 4°C in the presence of the following primary antibodies: ∼30 mg primary antibodies (1:2000 dilution in 2.5% BSA in TBS‐T) against mechanistic target of rapamycin (mTOR)^Ser2448^, total mTOR, ribosomal protein S6 kinase 1 (P70S6K1)^Thr389^, total P70S6K1, protein kinase B (Akt)^Ser473^, total Akt, eukaryotic translation initiation factor 4E‐binding protein 1 (4EBP1)^Thr37/46^, total 4EBP1, eukaryotic elongation factor 2 (eEF2)^Thr56^, total eEF2, ribosomal protein S6 (rps6)^ser240/244^, total rps6, Beclin‐1, ERK 1/2^Thr202/Tyr204^ (New England Biolabs, Hitchin, UK), Cathepsin L and calpain 1 (Abcam, Cambridge, UK). For nuclear proteins, immunoblotting was performed as above with muscle initially homogenized in RIPA buffer (Thermo Scientific) with 1 mm EDTA, 1 mm activated Na_3_VO_4_ (Sigma‐Aldrich) and a complete protease inhibitor cocktail tablet (Roche, Burgess Hill, UK). Primary antibody concentrations were 1:2000 for retinoblastoma (pRB)^S780^ (New England Biolabs), upstream binding factor 1 (UBF1)^S484^, total upstream binding factor 1 (UBF1), total transcription initiation factor 1 (TIF1a) (Abcam, Cambridge, UK) and 1:1000 for C‐Myc (New England Biolabs) and transcription initiation factor 1 (RRN3)^S649^ (Abcam). Membranes were washed 3 × 5 min in TBS‐T, incubated in horseradish peroxidase (HRP)‐conjugated secondary antibody (New England Biolabs; 1:2000 in 2.5% BSA in TBS‐T) at room temperature for 1 h, before the last 3 × 5 min washes in TBS‐T. Membranes were exposed to Chemiluminescent HRP Substrate (Millipore Corp., Billerica, MA, USA) for 5 min and bands quantified by Chemidoc MP (Bio‐Rad, Hemel Hempstead, UK). Software measures were taken to prevent pixel saturation. Protein‐loading anomalies were corrected to Coomassie protein (Welinder & Ekblad, 2011); relative arbitrary units (RAU) were normalized to Coomassie‐stained membranes and subsequently normalized to the rest leg at that specific time point. Plasma levels of testosterone, IGF1 and myostatin were analysed in all O subjects and nine Y subjects using enzyme‐linked immunosobent assays (ELISA) according to the manufacturer's protocol with the following commercially available kits: GDF‐8/Myostatin Quanitkine ELISA Kit (DGDF80, R&D Systems, Abingdon, UK), Testosterone ELISA Kit (Abcam ab108666) and IGF1Quantikine Elisa Kit (R&D DG100).

### Gene expression analysis

Total RNA was isolated by homogenizing 5–10 mg of muscle in 200 μl of TRizol (Life Technologies/Thermo Fisher Scientific) using two stainless steel beads (Tissue Lyser II, Qiagen, UK) for 1 min at 30 s^–1^. Samples were placed at ambient temperature for 10 min before 80 μl of chloroform was added and samples vortexed and incubated at ambient temperature for 10 min. After centrifugation at 12,000 *g* for 15 min at 4°C the upper aqueous layer was removed and RNA precipitated with an equal volume of isopropanol, incubation at room temperature for 10 min and subsequent centrifugation at 7000 *g* for 10 min at 4 °C. The pellet was washed twice with 1 ml of 80% ethanol, dissolved in 22 μl of RNA‐free water and quantified by spectrophotometry (NanoDrop Lite). For RT‐qPCR 500 ng of total RNA was reversed‐transcribed with the high‐capacity cDNA reverse transcription kit (Life Technologies) according to the manufacturer's protocol. Resulting cDNA was diluted 1:5 and 1 μl was added per well of 384‐optical well plates (Life Technologies). Exon specific primers were mixed with SYBR Select Master Mix (Life Technologies) and 11 μl of master‐mix was added to each well, with samples run in triplicate. Thermal cycling conditions were 2 min at 50°C followed by 2 min at 95°C and 40 cycles of 15 s at 95°C and 60 s at 60°C on a ViiATM 7 Real‐Time PCR System (Life Technologies). To control for RNA input, peptidylprolyl isomerase A levels were measured and target mRNA expression was quantified using the ΔΔ*C*_t_ method (Schmittgen & Livak, 2008).

### Statistical analyses

Descriptive statistics were produced for all data sets to check for normal distribution (accepted if *P* > 0.05) using a Kolmogorov–Smirnov test. All data are presented as means ± SEM. Subject physical characteristics were measured via Student's *t*‐test and all other data sets were analysed by repeated measures two‐way ANOVA with a Bonferroni correction using Prism v. 5 (GraphPad Software, La Jolla, CA, USA). Correlations were assessed using Pearson's product moment correlation coefficient. The α level of significance was set at *P* < 0.05.

## Results

### Subject characteristics

Subject baseline characteristics are shown in Table 1. The significantly different physical characteristics were skeletal muscle index (SMI), TFFM, 1‐RM and MVC, which were lower in O *vs*. Y subjects. Additionally O subjects had lower circulating concentrations of testosterone and insulin‐like growth factor 1 (IGF‐1), with no difference in myostatin levels. Only young subjects significantly increased testosterone levels after the first bout of RET. Monitoring of habitual dietary and activity behaviours revealed that during the study older subjects registered on average less activity counts per day and tended to have a decreased protein intake (g (kg fat free mass (FFM))^–1^ day^−1^).

**Table 1:** Subject characteristics

	Young	Old
Age (years)	23 ± 1	69 ± 1*
Height (m)	1.80 ± 0.02	1.75 ± 0.02
Weight (kg)	76 ± 3	80 ± 3
BMI (kg m^−2^)	23.6 ± 1	25.8 ± 1
FFM (kg)	62 ± 2	59 ± 2
ALM (kg)	27.7 ± 1	25.2 ± 1
ALM/h^2^ (ALM kg m^−2^)	8.5 ± 0.2	8.1 ± 0.1
SMI	33 ± 1	29 ± 1*
TFFM (kg)	6.0 ± 0.2	5.2 ± 2*
1‐RM (N)	546 ± 31	352 ± 31*
MVC (N m)	232 ± 20	172 ± 15*
Activity counts	72,584 ± 5746	52,445 ± 7462*
MET (min day^−1^)		
METs < 1.5	932 ± 65	932 ± 18
1.5 ≤ METs < 3	231 ± 32	314 ± 94
3 ≤ METs < 6	150 ± 30	149 ± 32
6 ≤ METs < 10.2	3 ± 1	4 ± 1
10.2 ≤ METs	1.3 ± 0.4	0.9 ± 0.4
Protein (g (kg FFM)^−1^ day^−1^)	1.73 ± 0.19	1.37 ± 0.09

Hormones	Rest	Acute RET	Rest	Acute RET
Testosterone (ng dl^−1^)	367 ± 19*	394 ± 20*†	274 ± 19	276 ± 18
Myostatin (pg ml^−1^)	4563 ± 403	4859 ± 450	3781 ± 373	3798 ± 424
IGF1 (ng ml^−1^)	155 ± 16*	158 ± 16*	84 ± 9	80 ± 9

Values are means ± SEM. ^*^Significantly different between young and old subjects *P* < 0.05, ^†^significantly different frm rest *P* < 0.05. 1‐RM, one repetition maximum; ALM, appendicular lean mass; FFM, fat‐free mass; MET, metabolic equivalent; MVC, maximum voluntary contraction; SMI, skeletal muscle index; TFFM, thigh fat free mass.

### Muscle strength, mass and architecture

**Figure 2:**
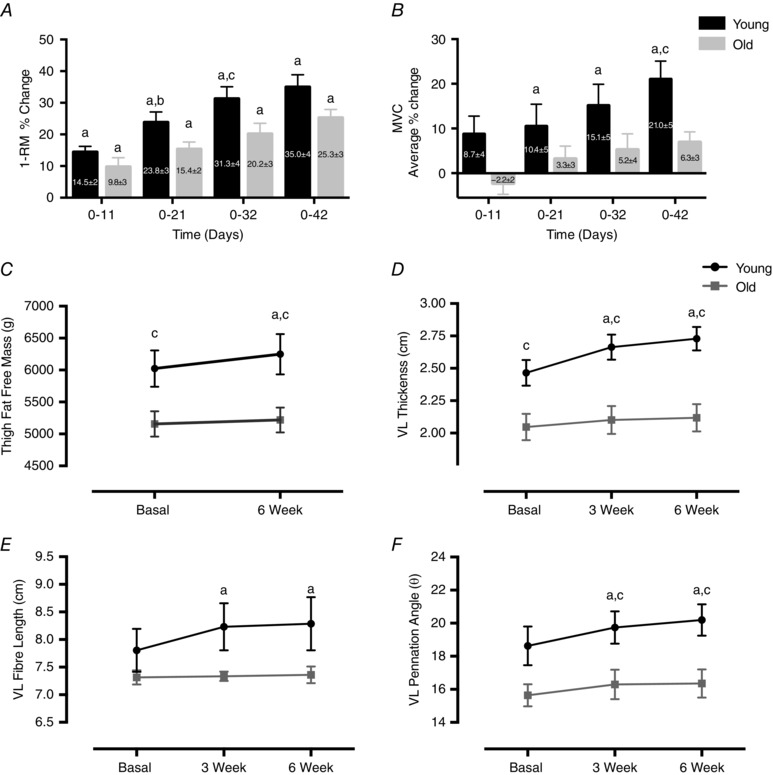
Muscle strength, mass and architecture Time course of changes in T legs as percentage change in 1‐RM from baseline (*A*), average percentage MVC from baseline (*B*), thigh fat free mass 0–6 weeks (*C*), VL MT (*D*), *L*_f_ (*E*) and θ (*F*). Values are means ± SEM. ^a^Significantly different from baseline, *P* < 0.05; ^b^significantly different from previous time point, *P* < 0.05; ^c^significantly different from old, *P* < 0.05. 1‐RM, one repetition maximum; *L*_f_, fibre length; MT, muscle thickness; T, trained; VL, vastus lateralis; θ, pennation angle.

Over the training programme (Fig. 1) Y and O subjects both increased 1‐RM, Y achieving 35.0 ± 4%, *P* < 0.01 and O 25.3 ± 3%, *P* < 0.01 after 6 weeks with no difference in the increase between groups (Fig. 2 *A*). In contrast, measures of MVC showed increases in Y only, with the average change across all joint angles at 6 weeks of 21 ± 5% (*P* < 0.01) in Y and 6.3 ± 3% in O (NS) (Fig. 2 *B*). However, taking the peak performance at any given angle for each individual (since optimal performance may vary by angle within individual subjects), both Y and O showed increased MVC (Y, 36.2 ± 5%, *P* < 0.001; O 16.4 ± 4%, *P* < 0.05). TFFM measured by DXA showed that in addition to having greater mass before and after RET, only Y increased TFFM after the 6‐week training programme, from 6023 ± 284 to 6249 ± 315 g (*P* < 0.05) *vs*. O, 5156 ± 198 to 5218 ± 194 g (Fig. 2 *C*). Temporal changes in VL architecture measured via ultrasound revealed by 3 weeks, RET induced increases in Y muscle thickness (MT, from 2.46 ± 0.1 to 2.66 ± 0.1 cm, *P* < 0.01; Fig. 2 *D*), pennation angle (θ, from 18.6 ± 1 to 19.7 ± 1 deg, *P* < 0.01; Fig. 2 *E*) and fibre length (*L*_f_, from 7.8 ± 0.4 to 8.2 ± 9 0.4 cm, *P* < 0.01; Fig. 2 *F*). By 6 weeks of training there was no further increase in any of these variables (2.72 ± 0.1 cm, 20.2 ± 1 deg and 8.3 ± 0.4 cm, MT, θ and *L*_f_, respectively). In contrast, O did not significantly increase any of these measures over the 6 weeks: MT, 2.0 ± 0.1 to 2.1 ± 0.1 and 2.1 ± 0.1 cm; θ, 15.6 ± 0.6 to 16.3 ± 0.8 and 16.4 ± 0.8 deg; and *L*_f_, 7.3 ± 0.1 to 7.33 ± 0.1 and 7.36 ± 0.1 cm at 0, 3 and 6 weeks, respectively. The untrained (UT) legs showed no changes in muscle architecture from baseline over the 6 weeks.

### Muscle RNA, DNA and protein concentrations

**Figure 3:**
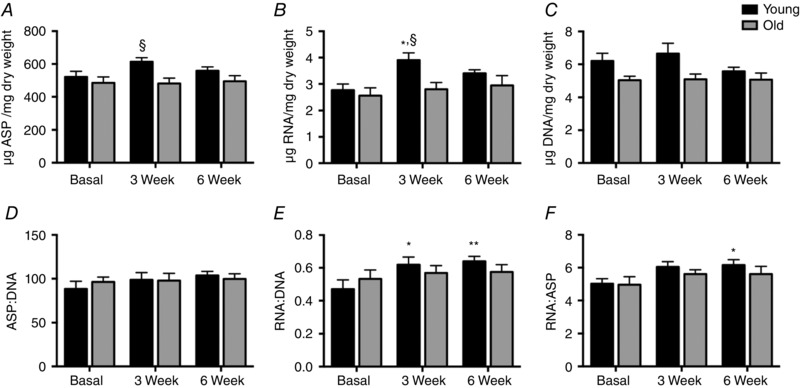
Muscle protein, RNA and DNA concentrations Time course of the changes in μg (mg dry weight muscle)^−1^: ASP (*A*), RNA (*B*) and DNA (*C*); and ratios of ASP:DNA (*D*), RNA:DNA (*E*) and RNA:ASP (*F*). Values are means ± SEM. Significantly different from baseline: ^*^ *P* < 0.05, ^**^ *P* < 0.01; significantly different from old: ^§^ *P* < 0.05. ASP, alkali soluble protein.

There were no changes in the alkali soluble protein (ASP) per wet weight muscle (μg mg^−1^) in trained (T) legs of either group throughout the 6‐week RET period, although Y had greater ASP at 3 weeks than O (613.5 ± 25 *vs*. 480.5 ± 33, *P* < 0.05; Fig. 3 *A*). The ratio of ASP:DNA (μg μg^−1^), a measure of cell size, displayed a trend to increase by 6 weeks (88 ± 8 to 103 ± 5, *P* = 0.1), whilst O showed no change at any time point (Fig. 3 *D*). RNA content (μg mg^−1^) increased in Y only at 3 weeks (2.75 ± 0.2 to 3.91 ± 0.2, *P* < 0.01) with a trend to increase at 6 weeks (3.38 ± 0.1, *P* = 0.1), whilst there was no change in RNA content in O (Fig. 3 *B*). The DNA content (μg (mg wet weight of muscle)^−1^; Fig. 3 *C*) did not change in either group over the training period; however, the ratio of μg RNA:μg DNA a reflection of ribosomal capacity per unit DNA, increased in Y at 3 weeks (0.47 ± 0.05 to 0.62 ± 0.05, *P* < 0.05) and remained increased at 6 weeks (0.64 ± 0.03, *P* < 0.01, Fig. 3 *E*). In contrast to Y, there was no change in RNA:DNA in O (0.53 ± 0.05 to 0.57 ± 0.05 and 0.58 ± 0.05, NS, at 0, 3 and 6 weeks, respectively). Finally the ratio of RNA:ASP (μg mg^−1^), primarily a measure of ribosomal capacity (Laurent *et al*. 1978; Waterlow *et al*. 1978), was elevated at 3 weeks (5.0 ± 0.3 to 6.0 ± 0.3, *P* = 0.06) and increased at 6 weeks (6.1 ± 0.3, *P* < 0.05) with no change in O (4.9 ± 0.5 to 5.7 ± 0.4 and 5.6 ± 0.5, NS) at 0, 3 and 6 weeks, respectively (Fig. 3 *F*).

### Muscle protein synthesis, absolute and fractional growth rates

**Figure 4:**
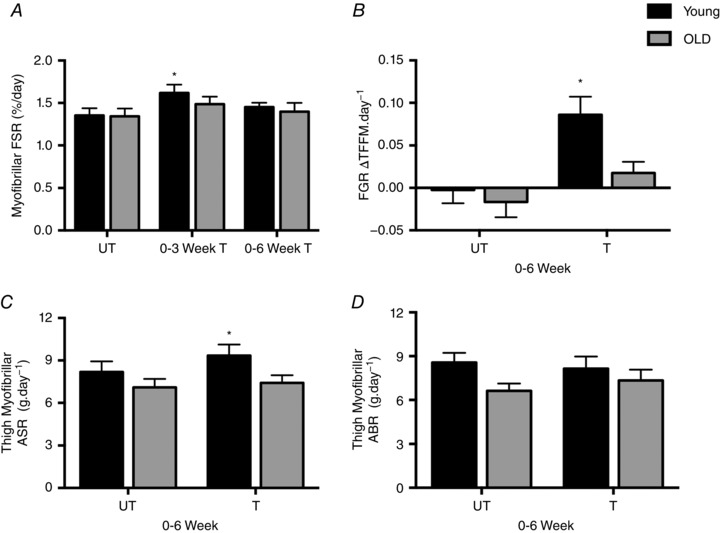
Muscle protein synthesis, absolute synthetic rate, fractional growth rate and absolute breakdown rate *A*, muscle protein synthesis (MPS) rates in UT and T legs in Y and O individuals. *B*, fractional growth rate (FGR). *C*, absolute synthetic rate (ASR). *D*, absolute breakdown rate (ABR). Values are means ± SEM. ^*^Significantly different from UT, *P* < 0.05. T, trained; UT, untrained.

There was no difference in muscle protein synthesis (MPS) in the UT leg between Y and O; 1.35 ± 0.08% day^−1^ and 1.34 ± 0.09% day^−1^, respectively. With the onset of RET, only Y increased MPS from 0 to 3 weeks (1.6 ± 0.01% day^−1^, *P* < 0.05 *vs*. O, 1.49 ± 0.08% day^−1^, NS); however, by 6 weeks MPS was no longer increased in Y (1.45 ± 0.05% day^−1^) and remained unchanged in O (1.39 ± 0.1% day^−1^; Fig. 4 *A*). Absolute synthetic rate (ASR; g d^−1^) was increased in the T leg only in the Y group over the 6 weeks (8.1 ± 0.1 to 9.3 ± 0.7; *P* < 0.05), with similar findings of increased fractional growth rate (FGR; % day^−1^) in Y only (–0.002 ± 0.016 to 0.086 ± 0.02, *P* < 0.001; Fig. 4 *B* and *C*). In contrast there was no change in absolute breakdown rate (ABR; g d^−1^) observed with either group over the 6‐week RET period (Y, 8.5 ± 0.6 to 8.1 ± 0.8; O, 6.6 ± 0.5 to 7.3 ± 0.7 g; both NS; Fig. 4 *D*).

### Intramuscular mRNA expression and signalling

**Figure 5:**
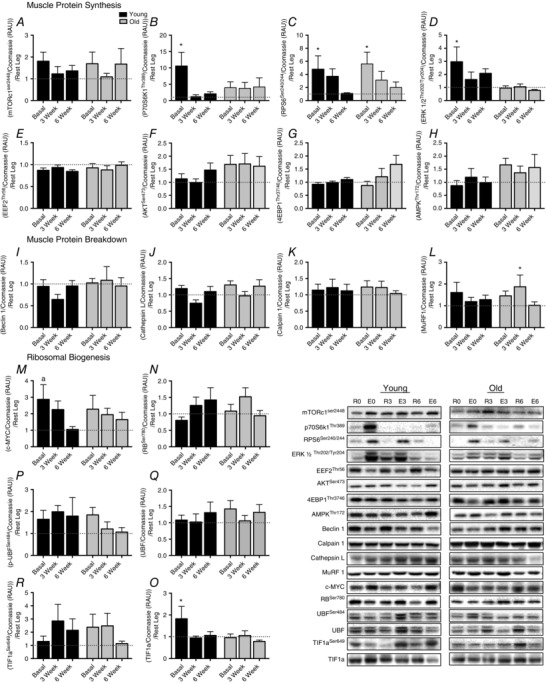
Intramuscular signalling Relative change compared with UT in mTORc1^Ser2448^ (*A*), p70S6K1^Thr389^ (*B*), rps6^ser240/244^ (*C*), ERK1/2^Thr202Tyr204^ (*D*), eEF2^Thr56^ (*E*), Akt^Ser473^ (*F*), 4EBP1^Thr37/46^ (*G*), AMPK^Thr172^ (*H*), Beclin‐1 (*I*), Cathepsin L (*J*), calpain 1 (*K*), MuRF1 (*L*), c‐MYC (*M*), pRB^Ser780^ (*N*), TIF1a (*O*), UBF1^Ser484^ (*P*), UBF1 (*Q*), TIF1a^Ser649^ (*R*). Values are means ± SEM. ^*^Significantly different from UT, *P* < 0.05. UT, untrained.

There were no differences between Y and O in the baseline expression of any of the genes measured (Table 2). However, 60–90 min after the first exercise bout Y showed increases in *c‐MYC* expression (*P* < 0.05), indicating activation of ribosomal transcription (*P* < 0.05). Additionally, only O increased *MAFBx* and *RPS26* 60–90 min after the first exercise bout in T legs compared to UT (*P* < 0.05) and showed a trend for increase with *MuRF‐1* (*P* = 0.06). Muscle anabolic signalling 60–90 min (Fig. 5) after the first exercise bout showed there were significant increases compared to UT in phosphorylation of P70SK1^Thr389^, RPS6^ser240/244^ and ERK^Thr202/Tyr204^. Further, only Y increased total levels of c‐MYC and TIF1a after the first exercise bout, whilst O increased phosphorylation of RPS6^ser240/244^ after the first exercise bout and MuRF‐1 60–90 min after RE at 3 weeks.

**Table 2:** Muscle mRNA expression

		Basal		3 weeks		6 weeks	
		Rest	Acute RET	Rest	Acute RET	Rest	Acute RET
C‐MYC							
5’‐GGTAGTGGAAAACCAGCAGCC‐3’	Y	1.00 ± 0.4	4.01 ± 1.6*	1.52 ± 0.6	2.45 ± 0.6	1.16 ± 0.3	2.49 ± 0.5
5’‐TCTCCTCCTCGTCGCAGTA‐3’	O	0.75 ± 0.3	1.89 ± 0.8	0.66 ± 0.2	2.95 ± 0.9	0.73 ± 0.2	2.73 ± 0.7
POL1RA							
5’‐CCTCAAGGTATCGCCCAGTC‐3’	Y	1.00 ± 0.1	1.41 ± 0.2	1.25 ± 0.2	1.49 ± 0.2	1.41 ± 0.2	1.03 ± 0.2
5’‐GGCAACTTCTGTTCTTGGGC‐3’	O	1.21 ± 0.2	1.36 ± 0.2	0.95 ± 0.1	0.97 ± 0.2	0.81 ± 0.1	1.41 ± 0.3
POL1RB							
5’‐TACTGTGCAACTTGGGGGTC‐3’	Y	1.00 ± 0.2	1.62 ± 0.4	1.93 ± 0.4	1.39 ± 0.3	1.71 ± 0.3	1.39 ± 0.2
5’‐GAGAATCTGCGATGCCTGGA‐3’	O	1.24 ± 0.4	1.22 ± 0.3	1.47 ± 0.5	1.69 ± 0.5	1.33 ± 0.3	2.60 ± 0.6
TAF1A							
5’‐AGGTTTAGCGCCTGCTCATA‐3’	Y	1.00 ± 0.3	1.62 ± 0.4	1.47 ± 0.3	0.84 ± 0.2	1.21 ± 0.1	0.69 ± 0.1
5’‐CTGAAATCACTCATACCCGCCT‐3’	O	1.14 ± 0.3	1.28 ± 0.4	1.27 ± 0.3	1.44 ± 0.3	1.55 ± 0.6	2.01 ± 0.6
TIF1A							
5’‐CATTTTGTGCCTCCCCGAGT	Y	1.00 ± 0.3	1.43 ± 0.5	1.61 ± 0.5	0.87 ± 0.3	1.61 ± 0.3	0.98 ± 0.3
5’‐GTATTGGCATGAGAAACCACGG‐3’	O	1.29 ± 0.4	1.25 ± 0.5	1.04 ± 0.2	1.18 ± 0.3	1.49 ± 0.4	1.57 ± 0.4
UBF							
5’‐AAGAAGCCTCCCATGAACGG‐3’	Y	1.00 ± 0.2	1.59 ± 0.4	1.34 ± 0.2	1.77 ± 0.4	1.51 ± 0.2	1.79 ± 0.6
5’‐CGGCCAGCTTTTTGTAGTGC‐3’	O	1.42 ± 0.4	2.00 ± 0.6	1.98 ± 0.6	0.69 ± 0.2	1.58 ± 0.3	3.03 ± 0.6
MuRF1							
5’‐GTGTTTGGGGCTCACCAGGC‐3’	Y	1.00 ± 0.2	2.86 ± 0.8	0.67 ± 0.1	1.61 ± 0.4	1.22 ± 0.5	1.01 ± 0.4
5’‐ACCTGGTGGCTATTCTCCTTGGT‐3’	O	1.41 ± 0.3	3.86 ± 1.5	1.01 ± 0.3	1.81 ± 0.5	1.74 ± 0.9	2.43 ± 0.8
MAFbx							
5’‐CTTTCAACAGACTGGACTTCTCGA‐3’	Y	1.00 ± 0.2	2.05 ± 0.4	1.19 ± 0.3	2.07 ± 0.3	1.47 ± 0.2	1.26 ± 0.2
5’‐CAGCTCCAACAGCCTTACTACGT‐3’	O	1.26 ± 0.2	3.12 ± 0.9*	1.20 ± 0.2	2.23 ± 0.7	1.38 ± 0.4	2.12 ± 0.6
20S proteasome							
5’‐CGTTTTCAACGGAGGTACTA‐3’	Y	1.00 ± 0.31	1.31 ± 0.4	1.36 ± 0.3	1.10 ± 0.3	1.41 ± 0.3	1.13 ± 0.2
5’‐TCAGCGTAAGACAGTCTCCA‐3’	O	1.21 ± 0.22	1.47 ± 0.5	1.18 ± 0.3	1.27 ± 0.3	1.69 ± 0.5	1.94 ± 0.6
Caspase 3							
5’‐TCCACAGCACCTGGTTATTATTC‐3’	Y	1.00 ± 0.3	1.45 ± 0.4	1.38 ± 0.3	1.00 ± 0.2	1.73 ± 0.4	1.52 ± 0.4
5’‐GCGTCAAAGGAAAAGGACTC‐3’	O	0.79 ± 0.2	0.91 ± 0.3	1.09 ± 0.3	1.27 ± 0.3	1.27 ± 0.4	1.85 ± 0.6
Calpain L							
5’‐GCCAAGCAGGTGAAC‐3’	Y	1.00 ± 0.3	1.81 ± 0.8	1.53 ± 0.4	1.01 ± 0.2	2.35 ± 0.9	0.80 ± 0.2
5’‐TGAAGTCTCGGAATGACATC‐3’	O	1.39 ± 0.3	1.95 ± 0.7	1.82 ± 0.5	2.01 ± 0.6	2.11 ± 0.7	2.55 ± 0.7
RPS5							
5’‐ATCATCAACAGTGGTCCCCG‐3’	Y	1.00 ± 0.3	1.52 ± 0.7	1.24 ± 0.3	1.13 ± 0.3	1.22 ± 0.2	1.18 ± 0.2
5’‐AGATGGCCTGGTTCACACG‐3’	O	1.42 ± 0.4	1.24 ± 0.4	1.80 ± 1.0	1.05 ± 0.3	1.32 ± 0.4	1.68 ± 0.3
RPSA							
5’‐CCCTACTGAAGACTGGAGCG‐3’	Y	1.00 ± 0.2	1.07 ± 0.2	0.96 ± 0.2	0.78 ± 0.2	0.78 ± 0.1	0.81 ± 0.2
5’‐AGAGCCTATGCAAGAACAGCTT‐3’	O	0.77 ± 10.1	1.19 ± 0.4	0.75 ± 0.3	0.45 ± 0.1	0.65 ± 0.1	0.76 ± 0.1
RPS26							
5’‐CGAGCGTCTTCGATGCCTAT‐3’	Y	1.00 ± 0.1	0.97 ± 0.1	1.11 ± 0.1	0.97 ± 0.1	1.01 ± 0.1	0.87 ± 0.1
5’‐CAGGTCTAAATCGGGGTGGG‐3’	O	1.00 ± 0.1	1.48 ± 0.2*	1.16 ± 0.1	1.32 ± 0.2	1.04 ± 0.1	1.17 ± 0.2
RPS3							
5’‐GGAAGTTTGTCGCTGATGGC‐3’	Y	1.00 ± 0.1	1.23 ± 0.2	1.19 ± 0.2	1.27 ± 0.2	1.17 ± 0.1	0.92 ± 0.1
5’‐ACATTCTGTGTTCTGGTGGCT‐3’	O	1.22 ± 0.3	1.49 ± 0.3	1.26 ± 0.2	1.35 ± 0.2	1.21 ± 0.2	1.82 ± 0.3
RPL13A							
5’‐TAAACAGGTACTGCTGGGCCG	Y	1.00 ± 0.2	1.31 ± 0.2	1.52 ± 0.2	1.19 ± 0.2	1.37 ± 0.1	1.11 ± 0.2
5’‐CTCGGGAAGGGTTGGTGTTC	O	1.44 ± 0.4	1.31 ± 0.3	1.19 ± 0.3	1.31 ± 0.4	1.11 ± 0.3	1.53 ± 0.3
RPL11							
5’‐AGGGTCTAAAGGTGCGGGA‐3’	Y	1.00 ± 0.2	1.91 ± 0.5	2.19 ± 0.4	1.12 ± 0.3	1.73 ± 0.2	1.09 ± 0.4
5’‐AGTCCAGGCCGTAGATACCA‐3’	O	1.33 ± 0.4	1.55 ± 0.5	1.05 ± 0.3	1.31 ± 0.4	1.15 ± 0.3	1.95 ± 0.4
Androgen receptor							
5’‐TCAGCATTATTCCAGTGGATG‐3’	Y	1.00 ± 0.2	0.99 ± 0.2	0.99 ± 0.3	1.04 ± 0.2	1.82 ± 0.4	0.83 ± 0.1
5’‐GGAGCTTGGTGAGCTGGTAG‐3’	O	0.88 ± 0.2	1.15 ± 0.3	1.06 ± 0.3	1.45 ± 0.3	0.90 ± 0.2	1.21 ± 0.3
Myostatin							
5’‐GCTGCGCCTGGAAACAGCTC‐3’	Y	1.00 ± 0.2	1.42 ± 0.4	2.08 ± 0.5	1.17 ± 0.4	2.74 ± 1.1	1.39 ± 0.3
5’‐ATCAGTTCCCGGAGTGGAGGC‐3’	O	2.32 ± 0.5	1.96 ± 0.6	2.78 ± 0.6	2.01 ± 0.3	2.58 ± 0.9	1.72 ± 0.3

Data expressed as fold change from young (means ± SEM). Forward primer is the upper sequence and reverse primer lower sequence. ^*^Significantly different from UT leg at that time point. MAFbx, muscle atrophy F‐box; MuRF1, muscle RING finger 1; RPL, ribosomal protein large; RPS, ribosomal protein small; TAF1A, TBP‐associated factor 1A; TIF1a, transcription initiation factor 1; UBF, upstream binding factor 1. POL1RA, RNA polymerase 1 subunit A; POL1RB, RNA polymerase 1 subunit B.

### Correlations

**Figure 6:**
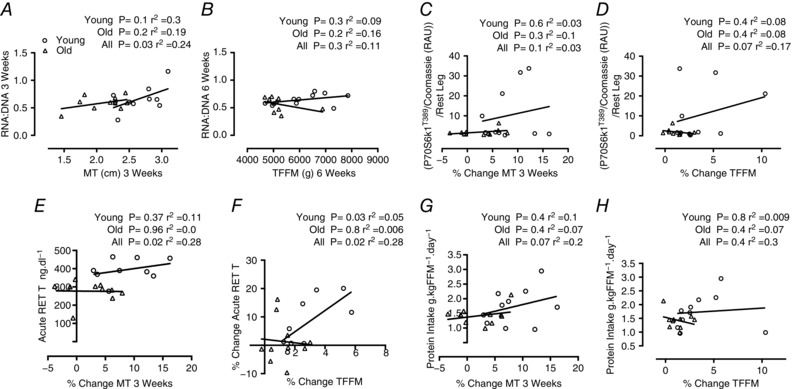
Correlations Correlations between RNA:DNA at 3 weeks and MT at 3 weeks (*A*), RNA:DNA at 6 weeks and TFFM at 6 weeks (*B*), the increase in P70S6K1^T389^ after first RET bout and the percentage change in MT at 3 weeks (*C*), the increase in P70S6K1^T389^ after first RET bout and the percentage change in TFFM at 6 weeks (*D*), testosterone after first RET bout and the percentage change in MT at 3 weeks (*E*), percentage change in testosterone after first RET bout and the percentage change in TFFM at 6 weeks (*F*), protein intake (g (kg FFM)^−1^ day^−1^) and percentage change in MT at 3 weeks (*G*), and protein intake (g (kg FFM)^−1^ day^−1^) and percentage change in TFFM at 6 weeks (*H*). MT, muscle thickness; RET, resistance exercise trained; TFFM, thigh fat free mass.

RNA:DNA was not correlated with MT in the UT state; however, at 3 weeks MT was correlated with the RNA:DNA content being driven primarily by changes in the young subjects (Fig. 6 *A*), although RNA:DNA was not correlated with TFFM at 6 weeks (Fig. 6 *B*). The change in P70S6K1 phosphorylation showed a trend for association with the change in TFFM across all subjects (*P* = 0.07) (Fig. 6 *D*), although it was not correlated with the change in MT at 3 weeks (Fig. 6 *C*). The acute RE‐induced levels of testosterone were correlated with the change in MT at 3 weeks (Fig. 6 *E*) and this change in acute testosterone levels was correlated with the change in TFFM at 6 weeks (Fig. 6 *F*). Protein intake across all subjects showed a trend for correlation with the early changes in MT at 3 weeks (Fig. 6 *G*, *P* = 0.07), although it was not correlated with the change in TFFM at 6 weeks (Fig. 6 *H*).

## Discussion

There has been a great deal of interest in recent years in uncovering the mechanisms underlying hypertrophic adaptations to exercise, and how these adaptations are influenced by age. Yet much of this information has been largely derived from ‘acute’ metabolic and molecular studies, which may not accurately reflect the true chronic adaptation. Therefore this study used novel D_2_O tracer techniques to investigate the temporal effects of RET on longer term MPS, hypertrophy and strength adaptations in groups of young and older individuals. This provides the first evidence that blunted hypertrophic responses in older individuals are underpinned by chronic deficits in MPS reflecting blunted ribosomal biogenesis and translational efficiency and lower anabolic hormone profiles, thus highlighting the multifactorial nature of the blunted hypertrophic responses.

Whether ageing impairs hypertrophic responses to RET remains contentious. For instance, RET‐induced hypertrophy of thigh muscles has been shown to be equal (Häkkinen *et al*. 1998 *b*; Mayhew *et al*. 2009) or impaired (Kosek *et al*. 2006; Greig *et al*. 2011; Mero *et al*. 2013) with age. In the present study, we utilized a 6‐week‐long progressive RET training programme during which we detected no significant increases in either TFFM (DXA) or hypertrophy‐related alterations in architecture (ultrasound) in old individuals indicative of blunted RET‐induced hypertrophy in thigh muscles, which are among the muscle groups more profoundly affected by ageing (Janssen *et al*. 2000) and most important for stability and preventing falls. Similarly, studies of an equal time period in older males have reported no increases in total lean body mass (Fragala *et al*. 2014) and similarly no change in VL muscle architecture or TFFM after 6 weeks RET (Scanlon *et al*. 2014). Indeed, even with long‐term (∼20 weeks) progressive RET, muscle gains are subtle, e.g. ∼1 kg in lean mass (Peterson *et al*. 2011), and diminished compared to younger counterparts (Phillips *et al*. 2012). Intriguingly, these data reflect the notion that RET‐induced hypertrophy occurs rapidly (first 3–4 weeks), diminishing thereafter (Brook *et al*. 2015). As such, long‐term stimuli are neither a prerequisite for muscle growth nor likely to significantly alter age‐related blunted responses. Thus, our present data support the prevailing notion of impaired hypertrophic responses to RET with ageing, although, with time, old individuals will see benefits in whole body strength (Peterson *et al*. 2010), and noticeable increases in 1‐RM and MVC were seen here after 6 weeks. Improvements in strength, whether dynamic or static, with little or no increases in muscle mass essentially derive from improvements in ‘muscle quality’. Whilst improvements in muscle quality are generally attributed to increased neural activity (Häkkinen *et al*. 1998 *a*; Tracy *et al*. 1999), improved strength may occur by many other modifiable aspects of muscle contraction (Fragala *et al*. 2015) improving physical function (Fiatarone *et al*. 1994; Fragala *et al*. 2014) and ultimately quality of life (Masel *et al*. 2009).

Skeletal muscle mass is controlled by the balance between MPS and MPB. Acute amino acid stable isotope tracer studies have shown no difference in fasting or resting MPS or MPB as a result of ageing (Volpi *et al*. 2001). The present study provides the first report of longer term MPS (over weeks) in young and older individuals in the same study under ‘basal’, conditions. The longer term D_2_O‐derived measures represent free‐living rates of MPS incorporating integrated responses to habitual nutritional intake and physical activity behaviours in young and older recruits. Since loss of muscle mass beyond ∼70 years of age occurs at a rate of 0.5–1% year^−1^ (and assuming no changes in MPB), daily MPS would only need to be decreased 0.0015–0.003% day^−1^ to account for such a loss. Thus, it is unsurprising that cumulative MPS (even over weeks) was not detectably modified by age. Instead, older muscle demonstrates blunted responses to anabolic stimuli key to regulating muscle homeostasis, i.e. nutrition and physical activity (Cuthbertson *et al*. 2005; Kumar *et al*. 2009 *b*). For example, the RET‐induced stimulation of MPS is attenuated (< 6 h) after a bout of RE in older *vs*. young subjects (Kumar *et al*. 2009 *b*). A similar result has been reported for responses to RET at 3, 6 and 24 h (Fry *et al*. 2011) with others also showing reduced rates in older individuals being sustained 24 h after a bout of RET (Mayhew *et al*. 2009). This study shows that findings from the acute setting translate into longer term deficits in MPS in the elderly, a notion that would not necessarily be obvious given that acute bouts of exercise (on which the foundation of anabolic resistance has been built) do not correlate to longer term hypertrophy (Mitchell *et al*. 2014). Finally, it is noteworthy that measures of MPS using D_2_O incorporate behavioural activities outside of study control, i.e. habitual physical activity and dietary behaviours. Although the older subjects registered fewer activity counts in this study, they were more active than an average population of their age group (Schrack *et al*. 2014) and additionally spent equal time in physical activities requiring the same level of metabolic equivalent (MET) as younger recruits. Studies where drastic decrements in activity are enforced have negative effects on muscle (Olsen *et al*. 2008; Krogh‐Madsen *et al*. 2010; Breen *et al*. 2013); in contrast, our old subjects are likely to maintain lower levels of activity (Dipietro, 2001) in which we are unaware of any evidence that habitual ‘inactivity’ has deleterious consequences upon RET‐induced muscle hypertrophy. Habitual behaviours may differ both among individuals and across age. It cannot therefore be entirely ruled out that slight age‐related behavioural differences could contribute to anabolic resistance, in both acute experimental settings and the chronic accumulation of these over time. However, intervention studies would be needed to resolve these interesting concepts and may warrant further study.

One of the key facets regulating MPS is ribosomal activity (so called ‘translational efficiency’). The acute simulation of MPS by RE relies upon increased translational efficiency (Chesley *et al*. 1992; Drummond *et al*. 2009). Acute RE (and nutrient interactions) converge on the pleiotropic protein kinase mTORc1, with subsequent downstream signalling enhancing translation initiation and elongation, a pathway essential to acute RE‐induced stimulation of MPS (Drummond *et al*. 2009). We and others have previously shown that P70S6K1 signalling after RE is blunted in older individuals (Kumar *et al*. 2009 *b*; Fry *et al*. 2011). In the present study we were able to look at the temporal activation of mTORc1 signalling in young and older individuals. In doing so, we found that P70S6K1 increased only after the first bout of exercise and that this was only observed in the younger group, while RPS6 phosphorylation was increased in both young and older individuals, again only after the first bout. The increase in P70S6K1 activation displayed a trend to be correlated with the increase in TFFM, adding to previous associations between P70S6K1 activation and hypertrophy (Baar & Esser, 1999) and dysregulation in MPS responses with age (Kumar *et al*. 2009 *a*; Fry *et al*. 2011). Additionally, these findings are in agreement with the notion that hypertrophy occurs early and also that RE‐trained individuals display attenuations in P70S6K1 (Coffey *et al*. 2006; Gonzalez *et al*. 2015). The absolute onset of attenuated signalling responses is unclear, and acute stimulation of anabolic signalling responses may remain up until before the 3‐week time point, with the duration and pattern of these responses unknown. However, early transcriptional responses are quickly modifiable (Murton *et al*. 2014) and activation of key signalling proteins is attenuated with repeated exercise stimulus (Ogasawara *et al*. 2013). This suggests that with prolonged training these signalling pathways become refractory to loading accounting for reduced rates of hypertrophy with training progression. Nonetheless, while age‐related blunting was seen in P70S6K1, this was not a uniform response across signals following the first exercise bout, suggesting that blunted translational efficiency is one aspect of, but perhaps not the entire explanation for, blunted muscle hypertrophy that is seen in older individuals.

In addition to increasing translational efficiency, prolonged muscle overload produces increases in RNA content and therefore capacity for protein synthesis (Laurent & Sparrow, 1977; Kirby *et al*. 2015), a subject matter which has been the of interest in renewed research (Nader *et al*. 2014; Figueiredo *et al*. 2015, 2016; Kirby *et al*. 2015; Stec *et al*. 2015; West *et al*. 2016). Indices of synthetic capacity (RNA:protein and RNA:DNA ratios; Laurent *et al*. 1978; Smith *et al*. 2011) increased early into RET in young individuals, plateauing by 3 weeks RET, and furthermore these responses were blunted with age. Additionally, with 3 weeks RET, MT was correlated with indices of synthetic capacity (RNA:DNA), which was not evident in the UT state, suggesting early hypertrophy is supported by early increases in synthetic capacity. To gain insight into the molecular mechanism regulating ribosomal biogenesis, upstream signalling pathways controlling ribosomal DNA (rDNA) transcription such as c‐MYC, which controls many aspects of cell growth and ribosomal biogenesis, were investigated. c‐MYC gene expression and protein abundance were increased in young individuals but were blunted with age, which is in line with previous reports showing that markers of ribosomal biogenesis are reduced in older adults 24 h after a bout of RET and suggesting rDNA transcription is compromised with age (Stec *et al*. 2015). Successful rDNA transcription requires multiple transcription factors, including UBF and TIF1a, with their activities controlled by many phosphorylation sites and upstream signalling pathways (reviewed in Kusnadi *et al*. 2015). However, similar to previous studies, TIF1a increased only in young individuals ostensibly driving rDNA transcription (Stec *et al*. 2015 *a*). Together, our data point to impairments in the regulation of translational capacity in addition to the aforementioned translational efficiency.

Beyond mechano‐sensitive pathways, muscle mass and RET‐induced muscle hypertrophy are also regulated by hormones, e.g. testosterone, IGF‐1, myostatin, etc. (Bhasin *et al*. 1996; Barton‐Davis *et al*. 1998; Whittemore *et al*. 2003; Kvorning *et al*. 2006). Ageing negatively regulates the ‘anabolic’ environment with declining endocrine function resulting in chronic low levels of many hormones such testosterone and IGF‐1 (Leifke *et al*. 2000). Whilst myostatin negatively regulates muscle mass, growth hormone, IGF‐1 and testosterone, whose levels when enhanced, or restored in older age, act as powerful anabolic agents and can substantially increase muscle mass (Bhasin *et al*. 2001; Sattler *et al*. 2009; Neto *et al*. 2015). Compromised hormonal balances with age are therefore likely to impair exercise adaptations, yet the precise influence in early exercise adaptations is unclear. Upon examination of these, we found no difference in myostatin between young and old individuals, and this was not acutely affected by RE. Similarly, sensitive liquid chromatography tandem mass spectrometry methods have demonstrated that in males, serum myostatin acts as a homeostatic regulator of muscle mass rather than a cause of sarcopenia (Bergen *et al*. 2015), further adding to oppose evidence that myostatin is implicated in the age relate muscle mass loss (Yarasheski *et al*. 2002). Circulating levels of many anabolic hormones decrease with age, and we found significantly lower circulating levels of testosterone and IGF‐1; moreover only younger individuals increased testosterone after the first bout of RE. Prevention of testosterone production in young males through provision of a GnRH inhibitor has been shown to prevent RET‐induced hypertrophy (Kvorning *et al*. 2006), whilst blunted hypertrophy has previously been reported in older men displaying reduced RE‐induced increases in testosterone (Kraemer *et al*. 1999). This highlights impaired endocrine response in older age, yet whether a lack of acute hormonal responses contributes to diminished hypertrophy is unclear. Acute RE‐induced increase in testosterone was correlated with the increase in TFFM, and this was strongest in Y where testosterone had increased. Further, whole body RET in older subjects with low testosterone shows minimal (∼+0.6%) changes in fat‐free mass (FFM) after 12 months RET (Hildreth *et al*. 2013) while no change in FFM occurred in elderly males with low normal testosterone after 24 weeks RET (Kvorning *et al*. 2013). Both studies showed greater improvements in FFM when testosterone supplementation and RET were combined. Together with our data this demonstrates that chronic low levels of testosterone and possibly a lack of acute increases may play a significant role in attenuated hypertrophy with age.

In addition to transient increases in MPS with RE, MPB is also increased (Phillips *et al*. 1997), probably to remove old or damaged proteins. The protein ligases muscle RING finger 1 (MuRF1) and muscle atrophy F‐box (MAFbx) are markers of ubiquitin proteasome pathway activity, which is thought to be predominant in acute RE responses (Fry *et al*. 2013). However, after a single bout of exercise, expression of *MuRF‐1* and or *MAFbx* have shown no change (Greig *et al*. 2011; Fry *et al*. 2013), equal increases (Fry *et al*. 2013; Stefanetti *et al*. 2014) or a greater increase with age (Raue *et al*. 2007) and therefore much uncertainty exists within the literature. We found an initial increases in *MAFbx* expression and a tendency towards greater *MuRf1* expression that only occurred in our older volunteers. Our measures were made 60–90 min after RET, in comparison to ≥ 2 h in these other studies and so our results may highlight key early differences in MPB. Measures of the FBR 24 h after RET have shown no difference between young and old; however, whether there are any earlier differences is unknown (Fry *et al*. 2013). Nonetheless, despite greater initial increases in MPB markers with age, there was no increase in ABR, highlighting that lack of muscle hypertrophy in older age is likely to be predominantly driven by blunted MPS responses rather than increases in MPB.

We also need to acknowledge limitations within our study. The data are limited to changes between three time points and each individual's response may temporally differ across the study period. Further, biopsy timings within this study reflect acute responses to RE and how these adapt over time, and do not demonstrate how resting levels of gene and protein expression may change with training. Additionally, although D_2_O permits long‐term measures of MPS, these are highly integrated, incorporating everyday habitual regimes with RE responses. In this study, subjects followed their usual habitual physical activity (potential impacts as previously discussed) and diet, which we recorded. In this study, older subjects tended, i.e. non‐significantly, to consume lower protein levels than younger subjects, which could theoretically impact our findings since acute protein feeding coupled to RET enhances MPS (Bukhari *et al*. 2015). That said, results of protein supplementation with RET in ageing have been mixed, i.e. shown to enhance (Esmarck *et al*. 2001) or not affect (Verdijk *et al*. 2009) muscle hypertrophy, while the effects of habitual protein feeding behaviours in this context of RET, ageing and hypertrophy remain poorly defined. That said, since protein intake was not correlated with muscle mass changes in young, old or all subjects combined in our study, any slight differences in protein intake are highly unlikely to be responsible for the observed anabolic resistance. In conclusion we have shown that age‐related deficits in anabolic responses to RET are multifactorial, a product of attenuations in translational efficacy and capacity and unfavourable hormone profiles, culminating in reduced MPS and compromised overall hypertrophy.

## Additional information

### Competing interests

No conflicting interests.

### Author contributions

All experiments were performed at the Clinical, Metabolic and Molecular Physiology laboratories, Royal Derby Hospital, University of Nottingham. D.J.W., K.S., P.L.G., N.J.S. and P.J.A. carried out the conception and design; M.S.B., W.K.M., B.E.P. and D.J.W. performed the experiments; M.S.B., B.E.P., D.J.W., K.S. and P.J.A. analysed the data; M.S.B., B.E.P., D.J.W., K.S. and P.J.A. interpreted the results; M.S.B., B.E.P., D.J.W., K.S. and P.J.A. drafted the manuscript; M.S.B., B.E.P., W.K.M., D.J.W., K.S., N.J.S., J.N.L., P.L.G. and P.J.A. edited and revised the manuscript. All authors have approved the final version of the manuscript and agree to be accountable for all aspects of the work. All persons designated as authors qualify for authorship, and all those who qualify for authorship are listed.

### Funding

This work was supported by a grant from The Physiological Society (to P.J.A. and K.S.), a project grant from the Dunhill Medical Trust (R264/1112) (to K.S., P.J.A. and D.J.W.), and a Medical Research Council Confidence in Concept award (CIC12019; to P.J.A., P.L.G., N.J.S. and K.S.) D.J.W. is a Medical Research Council‐Arthritis Research United Kingdom (MRC‐ARUK) Centre‐funded postdoctoral research fellow, and M.S.B. is supported by a University of Nottingham and BBSRC DTP award. Equipment was funded through monies provided from an award by the MRC‐ARUK Centre to the Universities of Nottingham and Birmingham.
